# Lessons learned from redesigning public health medicines supply chain model in Uttar Pradesh, India

**DOI:** 10.3389/fpubh.2025.1588227

**Published:** 2025-07-25

**Authors:** Vasanthakumar Namasivayam, Manuj Purwar, Sushant Jain, Shivalingappa Halli, Jagdeesh Kumar, Vikas Gothalwal, Marissa Becker, James Blanchard, Ties Boerma, Ravi Prakash

**Affiliations:** ^1^Government of India, Union Territory of Ladakh, Leh, India; ^2^Department of Community Health Sciences, Institute for Global Public Health, University of Manitoba, Winnipeg, MB, Canada; ^3^India Health Action Trust, Lucknow, Uttar Pradesh, India; ^4^Uttar Pradesh Medical Supplies Corporation, Government of Uttar Pradesh, Lucknow, India

**Keywords:** supply chain, health systems, essential medicines, procurement, Uttar Pradesh

## Abstract

Uttar Pradesh (UP), the most populous state of India with 238 million people, has over 30,000 public health facilities. Ensuring the continuous availability of essential medicines across these facilities is a significant challenge. An audit conducted in 2017 indicated large gaps in the availability of essential medicines in public health facilities. This study describes the lessons from Tamil Nadu’s Medical Supplies Corporation (TNMSC) that were adapted to inform the redesign of the medicines supply chain model and processes of Uttar Pradesh’s Medical Supplies Corporation (UPMSC). We identified seven essential pillars for a successful public health supply chain system through a desk review and learnings from TNMSC. These included a stable list of essential medicines, warehouses, centralized procurement, a passbook system, quality control, centralized payment, and digital e-tracking to enable real-time inventory and procurement decisions. The system design established a clear responsibility matrix: UPMSC is responsible for ensuring the availability of all essential medicines in the district warehouses at all times. The facility in-charge is responsible for ensuring the availability of the required drugs at the facility. The facilities are notionally allotted a budget and have complete freedom to pickup medicines from the warehouse, as long as they remain within the budget available. Under these seven essential pillars, several key processes were undertaken to improve vendor participation, reduce vendor dependency, synchronize tenders for all essential drugs, and establish rosters for facilities to pick up drugs from the warehouse. These efforts led to an improvement in the availability of essential medicines from ~40% to ~100%, with an average of 275 medicines out of 287 medicines available per warehouse. Supply orders increased from $58 million to $112 million, and facilities consumption value increased from $38 million to $90 million by April 2024. However, challenges such as last-mile delivery and prompt payment to vendors remain. This paper underscores the importance of system design in the public health supply chain and may be useful for other Indian states and low- and lower-middle-income countries (LMICs) with a similar context.

## 1 Introduction

The WHO health systems framework states that equitable access to and use of essential medical products, vaccines, and technologies—of assured quality, safety, efficacy, and cost-effectiveness—must be ensured by a well-functioning health system (1). One of the key aspects of achieving these objectives is having an efficient system for procurement, supply, storage, and distribution (1). Achieving Universal Health Coverage (UHC) goals of equitable, quality health services relies on access to medicines and health technologies (2). In 1977, the WHO published the first model list of essential medicines to assist countries in the formulation of their own national lists (3). Essential medicines are those that satisfy the priority healthcare needs of the population (3). Government procurement and distribution of medicines should largely be limited to the essential medicine list (EML) (3). Essential medicines have to be available at all times, adequately, in the appropriate dosage forms, of good quality, and at affordable prices (4). Though the overall budget of medicines varies widely in different states of India (5), approximately 10% of the government health budget goes into procuring medicines in India (5). There are huge inefficiencies in the procurement, storage, and distribution of medicines, leading to issues in the availability of medicines (5). Medicines contribute to high outpatient expenditure, approximately 67% in the case of the public health system (6). Accessibility, availability, and affordability of good quality medicines at minimum out-of-pocket (OOP) expenditure are key functions of the public health system to protect the public from the rising cost of healthcare (7).

Uttar Pradesh (UP), with about 238 million people, is India’s most populous state, with approximately 78% of them living in rural areas (8). UP has a vast network of more than 30,000 varied public health facilities [Medical College (MC) hospitals, District Hospitals (DH), Special Hospitals (SH), Community Health Centers (CHC), Primary Health Centers (PHC), and Sub-Centers (SC)] spread across 75 districts and 820 sub-district units (blocks), managed by 2 departments [Department of Medical Health and Family Welfare (DoMHFW) and Department of Medical Education (DoME)] (8). However, only 14% of the outpatient care and 28% of hospitalization (excluding childbirth), are catered to by the public health system in UP (9). Medicine expenditure as a proportion of outpatient expenses in public sector health facilities of UP was 71% (6). Those seeking in-patient care in public health facilities of UP incur significant OOP expenses, with medicines accounting for over 50 and 38% of OOP expenses in rural and urban areas, respectively (9). The Comptroller and Auditor General’s performance audit report on hospital drug management during 2017 for Uttar Pradesh’s public health facilities summed up the situation as “the Government was unsuccessful in providing an unbroken supply of essential drugs to the patients in public health facilities as per its own prescribed Essential Drug List.” This would have led to significant out-of-pocket expenditures being incurred by the patients, especially the poor. The drug procurement process was riddled with systemic flaws and numerous instances of non-adherence to the drug procurement policy/orders issued by the government from time to time, consequently impacting the availability of quality drugs (7). Moreover, even for those covered by health insurance, most insurance schemes in India do not cover medicine expenses for outpatient care (9).

**Figure 1:**
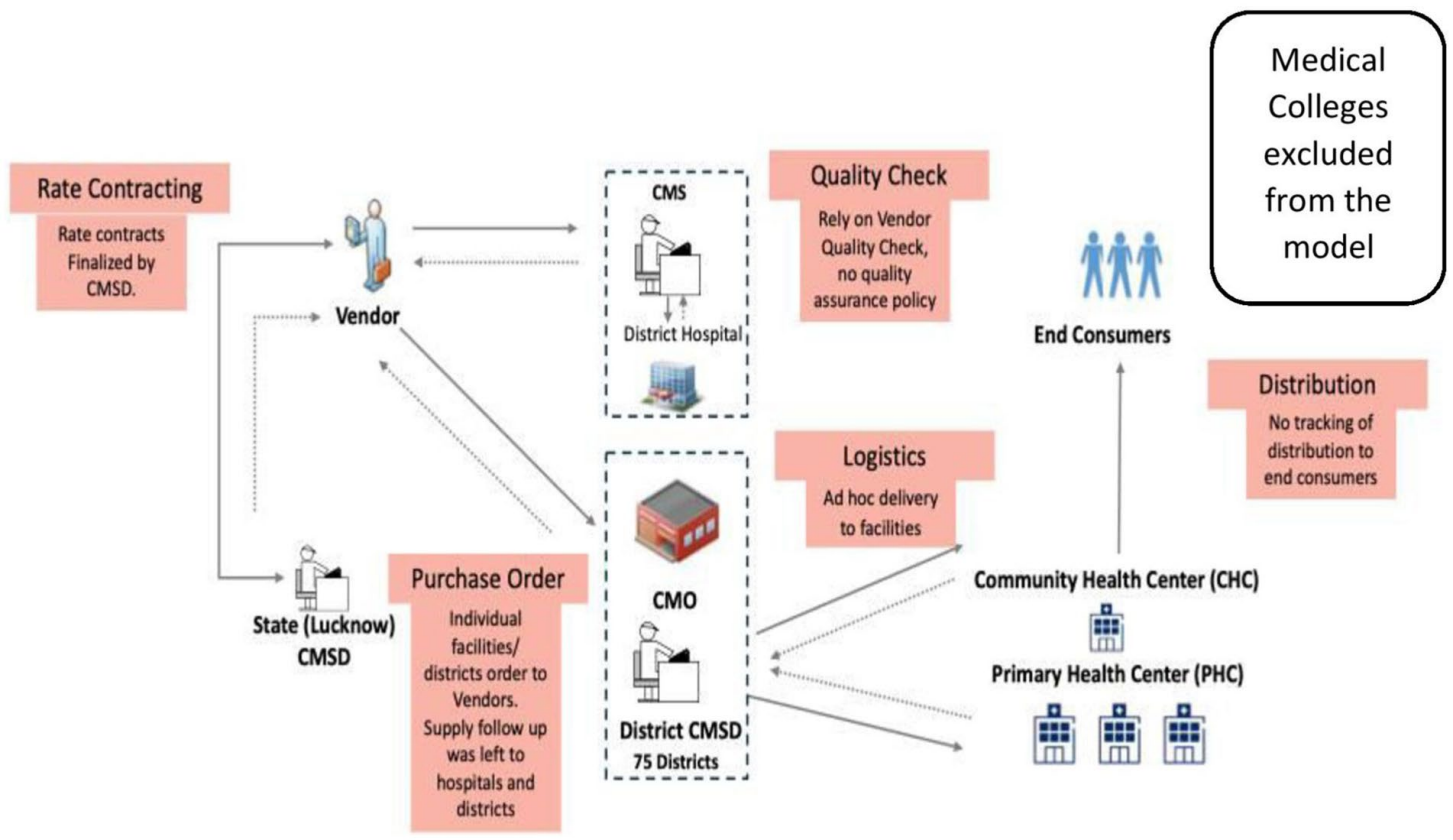
The public health supply chain model in UP-legacy model (CMSD).

The legacy model for drug procurement for the public health facilities of UP is depicted in Figure 1. The major gaps identified by the audit were that rate contracts (RCs) did not exist for all medicines, the bidders’ capacity was not analyzed, irregular procurement of medicines through local purchase, delayed/ non-supply of medicines, and minimal testing for quality, with no provisions for quality assurance (7). The audit report highlighted that in 2017–2018, rate contracts were available for only 18% of the 1036 drugs in the Essential Drugs List (EDL). Further, the district medical authorities—Chief Medical Superintendents (CMS) and the Chief Medical Officers (CMO)—were able to procure only 3 to 42% of the essential drugs. This is reflected in the availability of limited essential medicines, which ranged between 4 and 26% in District Women Hospitals and District Combined Hospitals (hospitals for both genders) and 7 to 42% in Community Health Centers where the study was conducted (7).

Even before the audit report was published, the Government of Uttar Pradesh (GoUP) was determined to strengthen the public health supply chain system. In 2015, GoUP hired Ernst & Young (EY) as an independent consultant through another project to assess the gaps in the public health medicines’ supply chain in UP. In their report titled “Procurement and Supply Chain Gap Analysis Report” (Unpublished document^1^), EY highlighted the following key gaps in the structure and processes. Some of these key gaps identified in the report were:

1. Inefficient management structure due to a highly decentralized structure, presence of multiple distribution channels, and inadequate capacity at the state and district levels
2. Inappropriate EDL design (lack of differentiation between essential medicine list and master drug list, contributing to an inordinately lengthy EDL)
3. Inadequate use of data/evidence for forecasting and the absence of a digital inventory management system
4. Poor store infrastructure and weak warehouse processes
5. Almost non-existent quality assurance/quality certification process (see footnote 1).

The Uttar Pradesh Technical Support Unit (UPTSU) also recommended to the GoUP in 2014 to create a separate entity (Unpublished document^2^), and the Government of Uttar Pradesh incorporated a new company named “Uttar Pradesh Medical Supplies Corporation” (UPMSC) in March 2018 (10, 11). However, though the federal and many state governments of India have set up similar separate entities (12, 13) for public health procurement, including medicines, they have produced mixed results in medicines’ procurement (14). Many states reported challenges in implementation, confounded by other systematic challenges such as how to do proper demand forecasting, reduce fragmentation of procurement sources, lack of technical competency of outsourced procurement agencies, suppliers’ non-compliance (quantity/quality/time frame), and absence of compliance checkpoints (15). Hence, there was a need to develop a good public health supply chain model for UPMSC to succeed.

## 2 Assessment of legacy system and redesigning of UP supply chain model

### 2.1 System design of the legacy supply chain model in UP (CMSD model)

As shown in Figure 1, the Central Medical Supply Department (CMSD), a cell located within the DoMHFW, UP, acted as the nodal intra-departmental agency for the procurement of medicines and other items required for the DoMHFW and the National Health Mission (an initiative undertaken by the government of India). CMSD’s primary role in the procurement of medicines was only finalizing an essential drug list (EDL) and maintaining the rate contracts (RCs) for the medicines under the EDL. The district’s Chief Medical Officers (CMOs) - head of all facilities within a district except DH and the Chief Medical Superintendents (CMSs) of DHs (75 CMOs, more than 100 CMSs in total) placed independent supply orders directly with the multiple vendors identified by the CMSD from time to time without taking advantage of the potential economies of scale. They also had limited power and means to ensure the performance of vendors, as the rate contract was with CMSD, and their influence was indirect. Most of the CMOs had limited storage spaces in comparison to the medicines’ requirement for a large number of facilities under their jurisdiction. Each of the medical colleges conducted independent tendering, placed supply orders directly with vendors, and operated outside the supply chain model of the CMSD. On the other hand, the suppliers were not getting unified, predictable, continuous work orders to plan supply, as each district/hospital followed different schedules. The multi-location payment claims process and settlement increased the cost of business due to delayed settlement. The suppliers were also self-certifying the quality of medicines by testing themselves in laboratories, with a potential conflict of interest. The supply of medicines from the district level to the facilities was ad-hoc and many a time not commensurate with the indent requirements. At the last mile, the doctors and pharmacists in the facilities who knew the requirements of the patients better had limited visibility of medicines’ availability at the district level and inadequate agency to get the drugs of their choice.

This manuscript presents the learnings from redesigning the public health supply chain model of UP, India. The aforementioned effort was supported by the Uttar Pradesh Technical Support Unit (UPTSU), operated by the University of Manitoba (UoM) to support GoUP achieve its health goals, in pursuance of a Memorandum of Cooperation (MoC) between the Government of UP (GoUP) and Bill & Melinda Gates Foundation (BMGF). UPTSU follows a “Program Science” approach, which is defined as “the systematic application of theoretical and empirical scientific knowledge to improve the design, implementation, and evaluation of public health programs.” (16) As a part of technical support on health systems strengthening, UPTSU recommended setting up an autonomous agency for medicines’ procurement and continues to support strengthening the company (UPMSC) to deliver its goals. The support areas of UPTSU to UPMSC have spanned areas such as setting up the company, system design changes, process improvements, recruitment of critical staff, identification of rental warehouses, rollout of information technology backbone, data analysis for decision-making, and risk assessment and mitigation. The Government of Uttar Pradesh (GoUP) led the reform process and change management from the legacy system. GoUP led the setting up of the UPMSC, posted reputed officers in leadership positions who championed the reforms, undertook the policy changes, system re-design, and processes improvement measures after due diligence of UPTSU recommendations, ensured funding support for the construction of warehouses through NHM, recruited procurement experts from the market, and continuously monitored the performance of UPMSC.

This paper consists of three different segments focusing on (i) the critical gaps identified in the legacy system, (ii) the system re-design changes, and (iii) key process improvements to deliver quality medicines to the public health facilities of UP. We also present data on some of the progress made so far by UPMSC in medicines’ procurement using the redesigned model, based on data from the Drugs and Vaccine Distribution Management System (DVDMS). Changes in the availability of medicines in the 75 district warehouses of UP over time have also been analyzed, and the data on the uptake of medicines by the facilities from the warehouses, and the quality assurance of medicines have been presented (17). The learnings presented here may help guide the strengthening of the public health medicines’ supply chain system in other States of India and low-and-middle-income countries (LMICs) experiencing similar challenges. For this, secondary data from DVDMS were used. None of the results presented in this paper are based on individualized data, and therefore, they do not compromise any individual’s identity.

### 2.2 Toward the redesign of the UP supply chain model

#### 2.2.1 Critical gaps in the legacy system

UPTSU studied various models for public health supply chain systems within India and globally to identify the critical gaps in the legacy system and suggest changes. The Tamil Nadu Medical Supplies Corporation (TNMSC), established in 1994 (18), is one of the long-standing, well-functioning public health supply chain system models in India. This model was studied in-depth to understand their policies, system design, processes, and responsibility matrix for adoption by GoUP. The TNMSC is a pioneer in the medicines’ procurement and distribution system in India (19). The success of the TNMSC lies in its centralized medicines’ procurement and distribution policy supported by a digitalized system of medicine management, among other factors (19). TNMSC has set up warehouses in all district headquarters for supplying medicines to facilities (19). There exists a passbook system where the virtual allotment of each facility is given in monetary terms so that hospitals can purchase different combinations using a given budget (20). They can obtain medicines from the approved list if funds are available in the passbook (20). The TNMSC also has a robust Drug Distribution Management (IT) System, which monitors the procurement and distribution of medicines (19). Receipts and issues of medicines are digitized in real time at the warehouse level, resulting in instantaneous stock adjustments (19). This is the basis of movement of medicines based on needs, thus avoiding shortages. The two-envelope system (separate technical and financial bid submission) of TNMSC ensures speedy and transparent procurement (19). Manufacturing units that have a good manufacturing practices (GMP certificate from the WHO) and with a minimum annual turnover were only provided the contracts for medicines’ supply (19). The TNMSC model’s success is also shown by the lower prices due to the competitive bidding and bargaining power (19).

The EY report mentioned above also highlighted the key gaps in the structure and processes of the legacy system. To get a deeper understanding of the model of TNMSC, visits were made by GoUP and UPTSU officials to help answer some important process-related questions, such as: How does TNMSC estimate the quantity to be procured? and How does the TNMSC make prompt payments to the suppliers?

It was learnt during the visit that while the annual demand estimation of medicines in UP is collected from the facilities and aggregated to be put in the tender documents for the discovery of prices, the TNMSC relied on ‘scientific forecasting’ based on past consumption data available with them, however they also collect the demand from the facilities. The quantities demanded were way off the mark in UP, leading to medicines’ non-availability on multiple occasions or oversupply of unnecessary medicines, as more quantities than required have been procured. While in the initial years of TNMSC operations, they used the disease profile and imperfect consumption data of medicines as a better yardstick than relying solely on the demand requests from the facilities for tendering requirements. The refilling of the warehouses was based on actual consumption. Regarding timely payment to the suppliers, it was learnt that while the states of India pay their vendors through a separate treasury system to which each of the bill is presented for clearance, TNMSC has evolved a unique mechanism called ‘PD’ account which provides them the necessary flexibility to pay the amount to vendors easily without the cumbersome treasury process, manage their cash flow well, and the annual earnings of TNMSC are calibrated to the actual expenses so that excess profit is not earned by the company, leading to higher taxes.

The document reviews, EY report, and TNMSC visit helped identify the critical gaps (mentioned above) in the legacy model of UP. UPTSU recommended system design changes and key process improvements based on the lessons learned to GoUP. GoUP, after due diligence, issued a Government Order dated 19 March 2019 on the redesigned model (UPMSC model) (Unpublished document^3^). Key process changes were also executed by UPMSC leadership through multiple internal orders.

#### 2.2.2 System re-design changes in UPMSC

**Figure 2:**
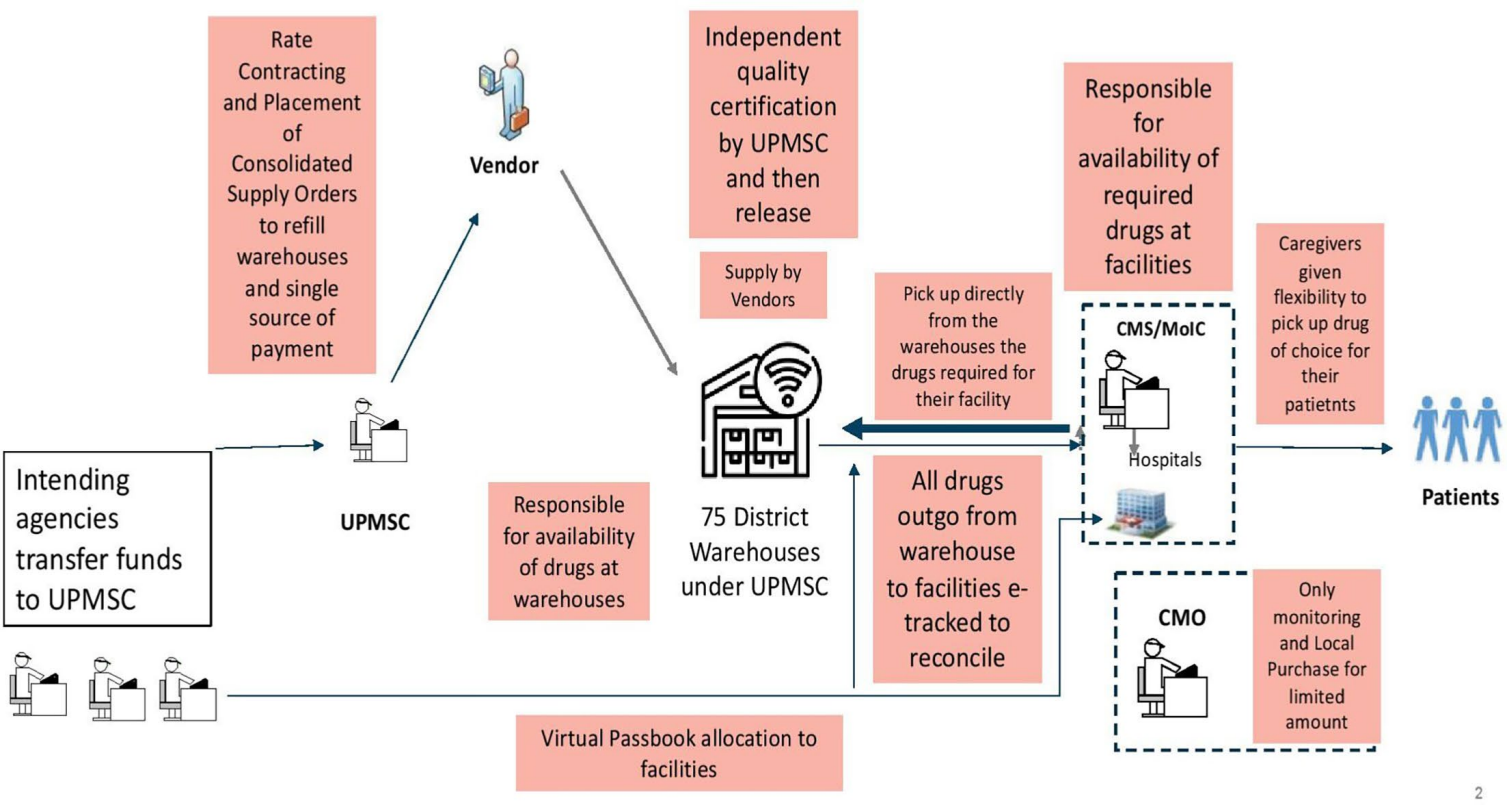
The redesigned UPMSC public health supply chain model in UP.

The redesigned public health supply chain model (UPMSC) is shown in Figure 2. UPMSC does the tendering to identify the rates for EDL, places supply orders centrally for all 75 warehouses’ requirements, does quality control of all batches of medicines supplied, captures the data of medicines outgo from warehouses to various facilities, and inputs of medicines received from suppliers in real-time. The redesigned model ensures that there is clear singular responsibility for activities in the entire value chain of medicines’ procurement, tendering, supply, quality assurance, fund transfer, payments, and uptake by facilities. The UPMSC is only responsible for making all EDL available in all 75 warehouses across 365 days. The health facilities come and pick up their drug of choice within the virtual budget (passbook) allocated to them by the health department. Facilities are responsible for drug availability in the facilities.

**Figure 3:**
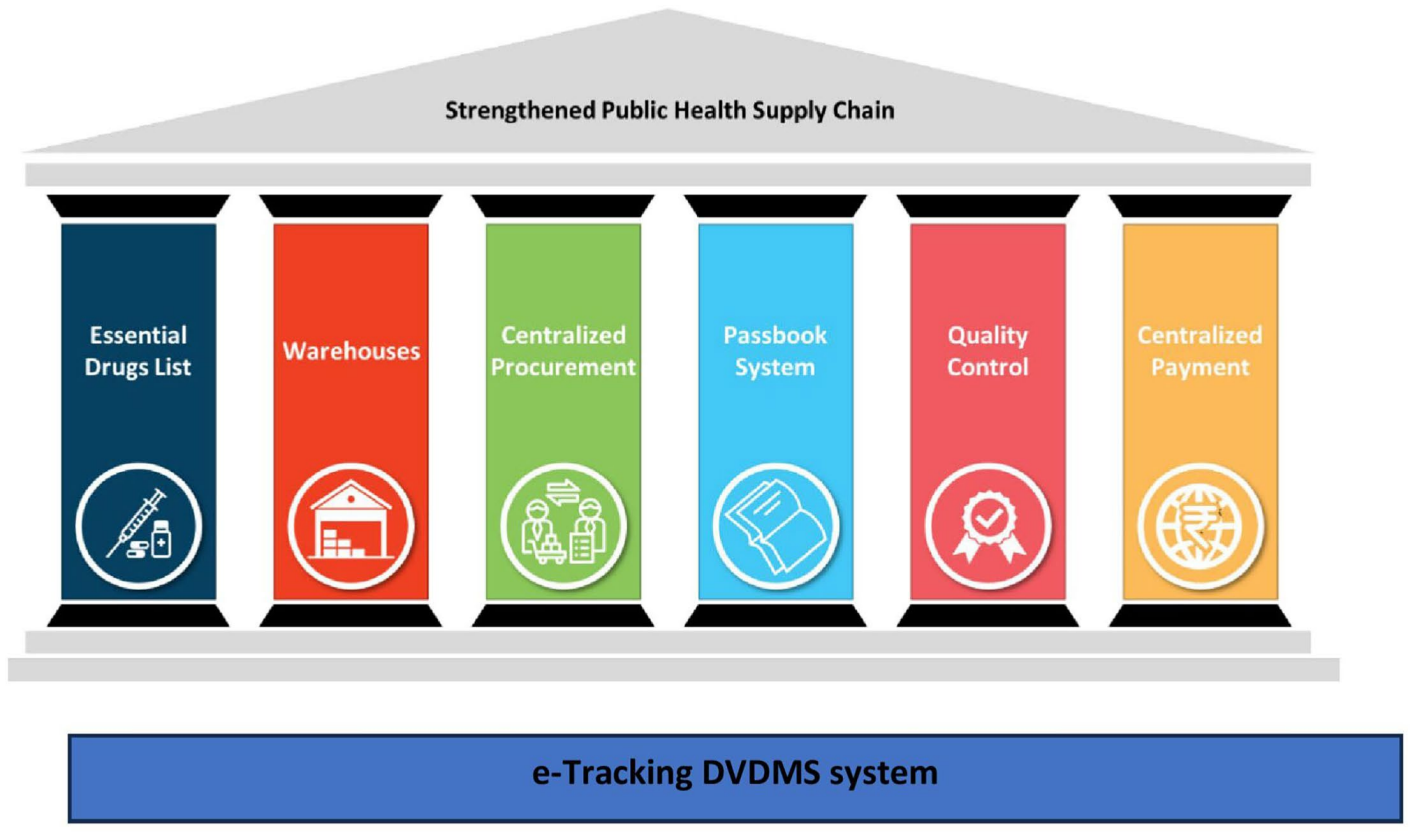
Seven essential pillars for an efficient medicine supply chain system.

During this process, UPTSU identified seven essential pillars (Figure 3) that are critical for the success of a public health supply chain model, which are elaborated in the discussion section.

#### 2.2.3 Key process improvements executed by UPMSC

Apart from the system re-design using the seven pillars, UPTSU also identified certain key process improvements that are required. The key process gaps identified in these seven pillars in the legacy model, compared with the TNMSC model and the changes done (UPMSC model), are given in Table 1.

**Table 1:** Differences in the key supply chain processes in the seven essential pillars between CMSD, TNMSC, and UPMSC.

Supply chain function	Legacy model (CMSD)	TNMSC model	UPMSC model
Pillar 1: EDL list	Very large essential drugs list (~1036 medicines)	EDL of around 300 medicines, maintained for a long period. Separate specialized drugs list (SDL) for medicines beyond EDL procured based on specific indents.	Essential drug list was reviewed and rationalized to identify the most critical medicines resulting in a list of ~300 medicines, which has since been increased to 393 with categorization for DH, CHC, PHC, and SC. Medicines beyond EDL are procured on an intended basis.
Pillar 2: District Warehouses for medicine storage, and distribution	Limited warehouse capacity catered by CMO/CMS stores, where vendors are directly supplied and these stores are controlled by CMOs/CMSs.	All district headquarters have warehouses under the control of TNMSC.	Warehouses in 61 districts on a rental basis and permanent warehouses in 14 districts with UPMSC, covering all 75 districts.
Pillar 3: Minimum Turnover criteria for vendors to participate in the tender.	Very high turnover, with the assumption that higher turnover companies have better quality.	Low turnover for less entry barriers for new vendors to participate in tenders, with emphasis on pre-inspection, strict double-blinded quality assurance.	Pre-inspection of suppliers unit is yet to be implemented, though agreed in principle by UPMSC and GoUP, due to manpower shortage. Strict double-blinded quality control measures were implemented.
Pillar 3: Vendor dependency	Rate contracts are limited to a single vendor for the entire state, and the medicine supply depends on the sole supplier’s performance.	Only a maximum of 60–80% requirement given to one vendor, and the remaining supply quantity distributed to vendors who match the lowest bid price. This led to the risk distribution of vendor performance.	Same as TNMSC. Rate contracts established for a 2-year period (longer period was established initially, which has now a 1-year period) with multiple vendors at the same price to reduce the risk of vendor non-performance.
Pillar 3: Tender process	No clear timelines fixed, leading to multiple tender processes throughout the year. Tender evaluation was an in-house closed process, and the results were published alone.	Clear timelines for floating and finalization of tender. Tender evaluation done in the presence of all vendors/representatives with video graphing, resulting in faster resolution of tender queries and closing of tenders.	TNMSC process adopted for faster evaluation of tenders and query resolution within a 4-week period. The time period can still be reduced to a large extent, as query resolution and finalization of tenders take a long time.
Pillar 3: Placement of supply orders	By more than 200 units in 75 districts, disaggregated and at variable times.	Auto-generated and modulated by centralized placement of supply orders. Emphasis on tracking supplies after tender finalization.	Auto-generation and modulated placement of supply orders by only one unit (UPMSC), aggregating the deficits below the mean stock level (MSL) in 75 warehouses
Pillar 4: Medicines flow to facilities	Though facilities could intend to obtain medicines from CMO stores, there was no visibility of medicines’ availability at CMO stores. There was also a ‘Push’ to take some medicines to the facilities, even if they are not needed, when more than the required quantities were supplied by the vendor.	The facilities are at liberty to take any of the medicines within the EDL and the budget available in the passbook. Days in the week were fixed for facilities to pick up medicines of their choice.	The facilities have complete freedom for intending and picking up EDL medicines of choice on a fixed day within the virtual budget available in the passbook.
Pillar 4: Passbook System	Absent	Budget funds are transferred to TNMSC by the different directorates and NHM. Directorates then virtually allocate the amount transferred to TNMSC to the facilities under their control, which is entered in the digital system.	Same as TNMSC. Dual entry passbook maintained at the warehouse and facility for easy reconciliation of funds allocated to UPMSC, and to monitor usage.^a^
Pillar 4: Transportation of medicines to facilities from the districts	Neither a separate budget nor a clear schedule.	Specific days to pick up medicines, budget, and the responsibility lies with the facilities.	Specific days, budget, and facilities to pick up the medicines required for them. (‘Pull system’). Release of this budget to the facilities for usage continues to be an issue.
Pillar 5: Quality control	Supplies were accepted based on vendor-provided quality testing certificates, with random sampling done by the drug controller.	Double-blinded NABL laboratory tests on all batches of medicines supplied by vendors by TNMSC and not for standard quality drugs referred to the drug controller.	Each batch of supplies is double-blinded, quality tested by NABL-certified laboratories, and then released for consumption to the facilities only when satisfactory.
Pillar 6: Centralized payment system	Decentralized at more than 150 places.	Centralized and prompt through the PD account system.	Centralized through the treasury system. There is scope for improvement in automated prompt payment systems and based on verifiable documents.
Pillar 7: e-tracking	Absent	Digital system that captures in real time the supplies and issues at the district warehouses linked to the entry in passbooks of the facilities.	Warehouse input and output to facilities are tracked through DVDMS in real-time.

^a^Unpublished document: Government Order. No 845 dated 17 July 2019 regarding implementation of passbook system for distribution of medicines in the state.

### 2.3 UPMSC performance so far

**Figure 4:**
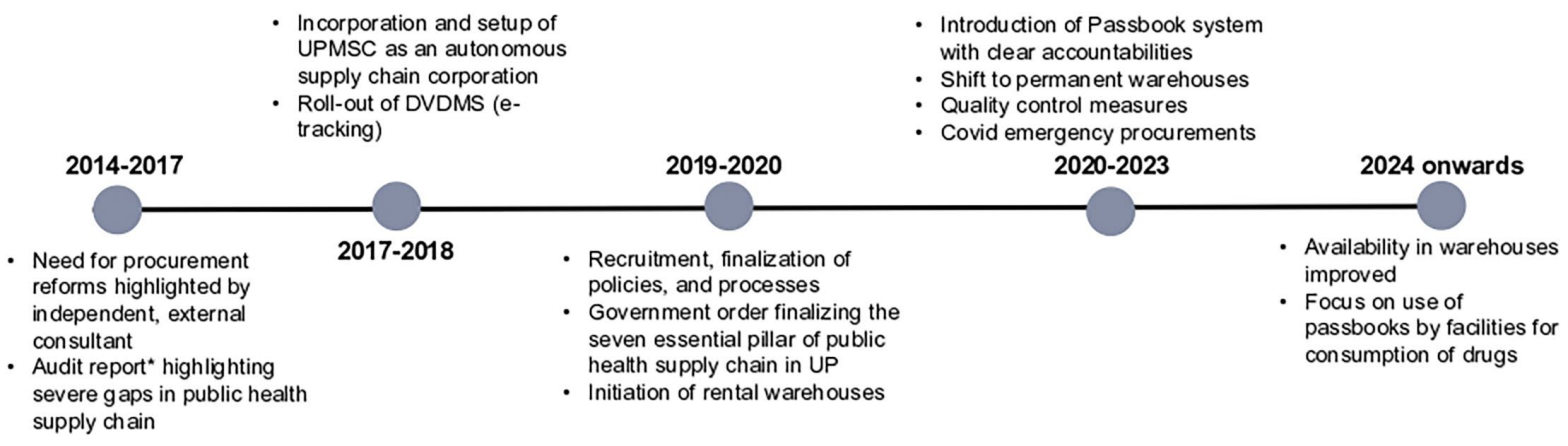
Timeline of system design and process reforms in UPMSC.

Below are some of the progress made by UPMSC in terms of making EDL medicines available in the 75 warehouses, values of purchase orders placed and consumption, and quality testing of medicines. The timelines of major reforms are depicted in Figure 4, which enables linkage between the reforms and performance.

**Figure 5:**
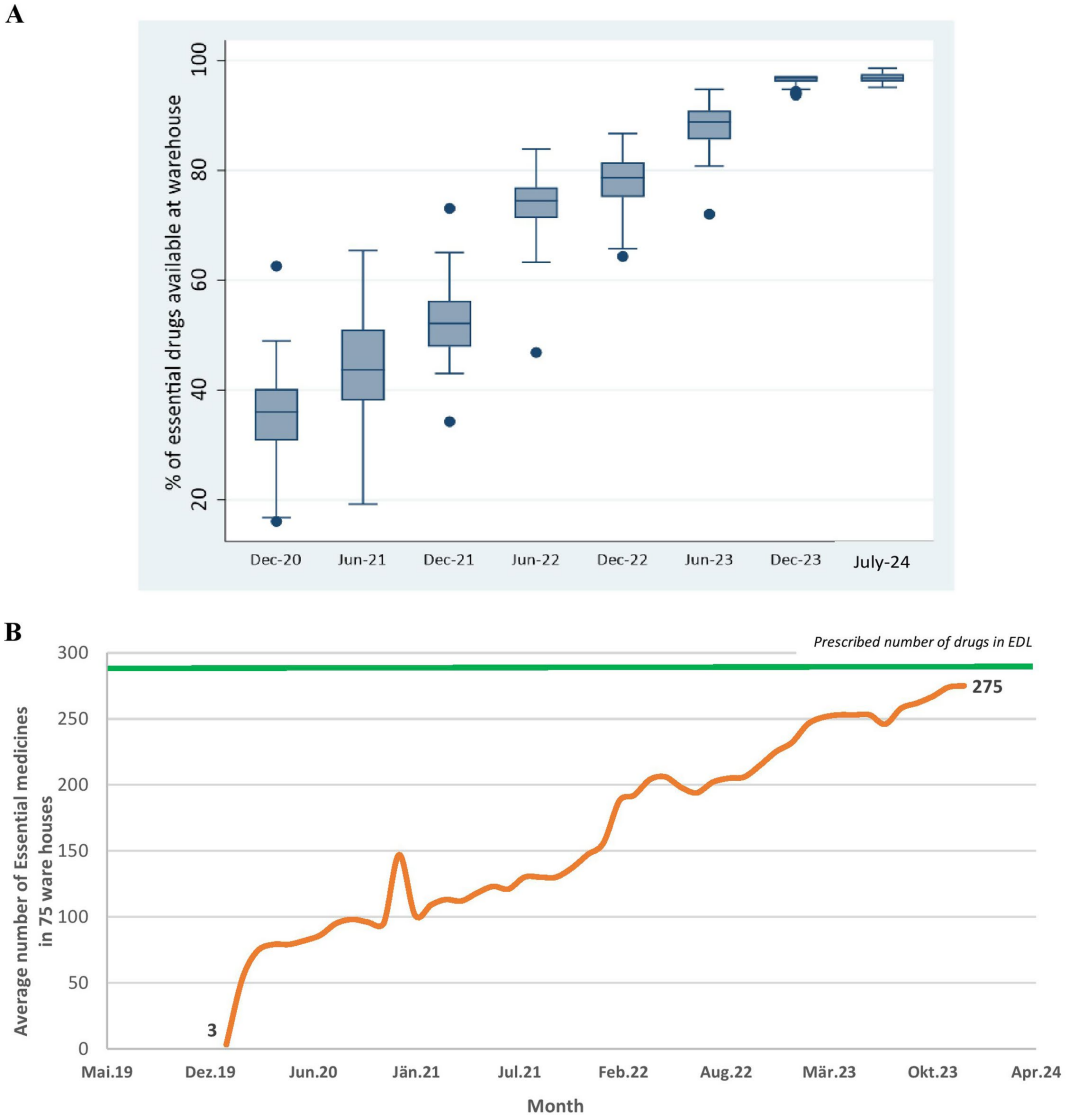
**(A)** Percentage of EDL medicines available across 75 district warehouses over time. **(B)** Average number of EDL medicines across 75 district warehouses overtime. DVDMS data.

Procurement related performance of UPMSC: Figures 5A,B shows that the overall availability of EDL medicines in all warehouses improved from 34 to 88% between December 2020 and July 2024, with a substantial reduction in heterogeneity in the availability of medicines between warehouses. Figure 5B shows that the average availability of EDL medicines in 75 warehouses increased from 27 to 97% between June 2020 and July 2024.

**Figure 6:**
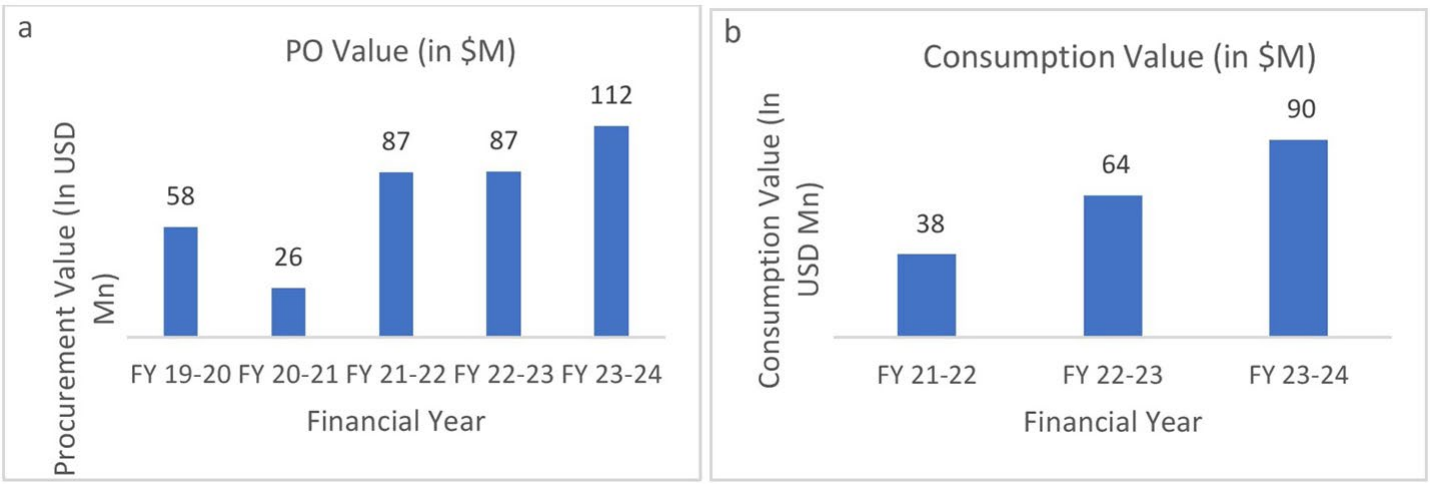
**(a)** Annual purchase order (PO) value by UPMSC. **(b)** Annual consumption value of medicines by facilities. Source: DVDMS data.

Supply order related performance of UPMSC: Figure 6A shows that the annual procurement value of medicines done by UPMSC recorded a two-fold increase from approximately 58 million USD to 111 million USD between 2019 and 2024. The dip in 2020 is due to the COVID-19 pandemic, where UPMSC played a key role in pandemic procurements. Figure 6B shows that commensurately, the consumption of medicines value due to uptake by the facilities increased from US$37 million to US$89 million between 2021 and 2024. The number and type of facilities picking up medicines from the UPMSC warehouses have increased substantially.

**Figure 7:**
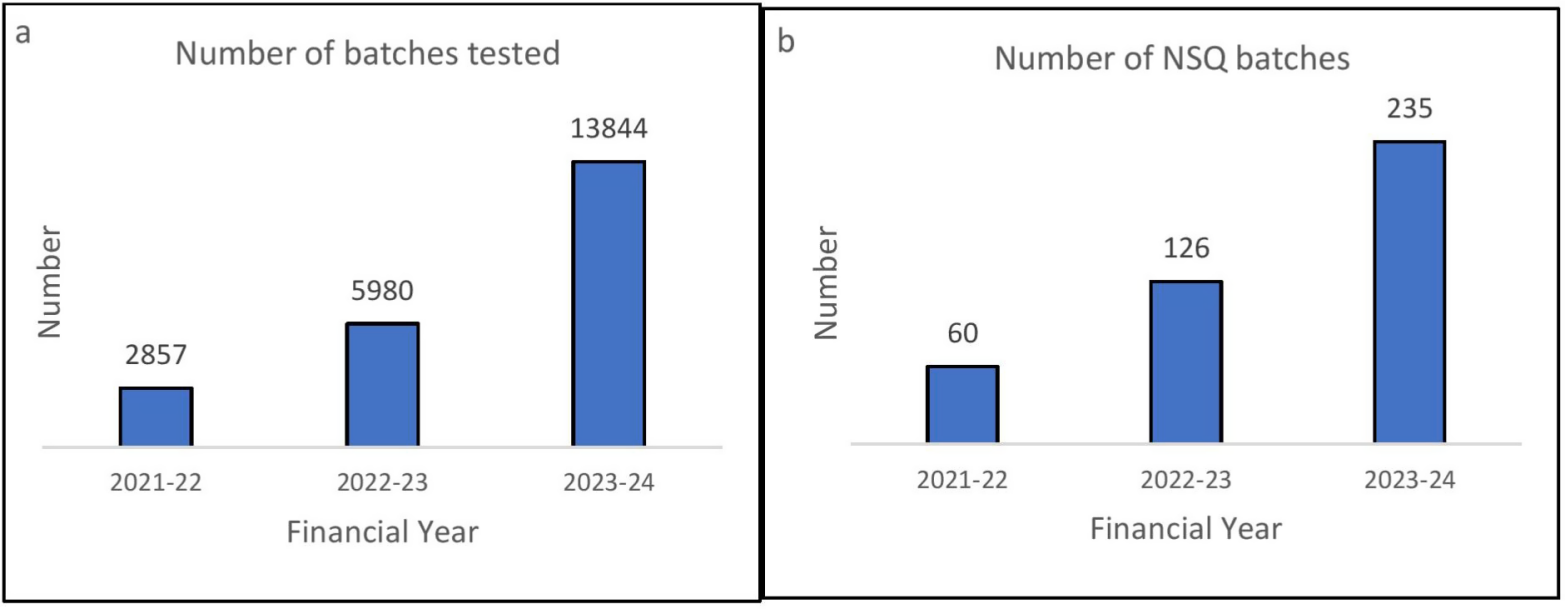
**(A)** Number of batches tested annually by UPMSC. **(B)** Number of not od standard quality (NSQ) drugs identified by UPMSC annually. DVDMS data.

Quality testing-related performance of UPMSC: Figure 7A shows that the number of batches tested for quality assurance increased from 2857 in 2021–2022 to 13844 in 2023–2024. Figure 7B shows that the number of non-standard quality (NSQ) medicines identified by UPMSC is also increasing annually, indicating that the quality control measure is functioning.

The above findings indicate a positive trend in the performance of UPMSC in medicines’ procurement, storage, quality testing, and delivery. The effect of this on increased public healthcare utilization and reduced OOP due to drugs on the population is yet to be evaluated.

## 3 Discussion

Essential medicines are one of the most cost-effective elements for any health system, with an immediate and long-lasting health impact (21). Despite rights to medicines enshrined in Sustainable Development Goal (SDG)-3, there remain some core problems with essential medicines in LMICs (21). The availability of essential medicines, particularly in the public sector, is still poor in many LMICs (21). Cost-effective, quality-assured medicines are not guaranteed to be available, prescribed, or used appropriately (21). There is much interconnectedness and many actors that influence and shape ‘medicines’, and to understand, a systems approach is required (21). Efficient public procurement systems are critical for ensuring access to medicines and enabling universal healthcare delivery. However, the essential medicine shortage in the Indian public healthcare system is significant and is exacerbated by inefficiencies in the procurement system (20). Whereas there are clear recommendations to include access to medicines and their appropriate use in achieving UHC as an explicit focus in health system strengthening and efforts toward universal health coverage, how to reduce LMICs current disproportionate spending of households’ and systems’ budgets on medicines, and how to achieve it despite good intentions is a challenge (21). Apart from scaling up funds for medicines’ purchase, optimum utilization of available resources is equally important (20), through a well-functioning public health supply chain model.

In India, one of the following arrangements is followed for public procurement by the central and state government institutions (18):

(i) Pooled procurement,
(ii) Central rate contract system,
(iii) Decentralized procurement, and
(iv) Local purchase.

The model followed by UP prior to 2017 was that of the central rate contract system, while TNMSC is a pooled procurement model. While literature search indicated some of the critical elements for TNMSC’s success, such as warehouses, passbook system, and IT system, these were generic in nature and would have been easy to adopt successfully by other states which operate in a similar context. Many states have not been able to emulate their success (15).

While redesigning the legacy model, UPTSU learned that while consultants do advocate for emulating certain successful private sector supply chain models and processes for public health medicines’ supply chain models, there are certain fundamental differences between the two. These differences should be understood for system re-design and adoption. The profit motive in private sector supply chain aligns intentions and incentives through the chain of command from shareholders to managers to employees, whereas in the public health medicines’ procurement, the intentions and incentives of actors such as political executives, senior health administrators, district managers, and doctors’ may not be aligned, as profit is not the singular motive or incentive. Furthermore, in general, the private sector entities operate facilities that are of a similar nature and size compared to those of the public sector, which operates hospitals at the tertiary care level to those of a sub-center providing primary healthcare with varying medicine requirements. The choice of access to hospitals and medicines for customers is broader in the private sector compared to that of poor patients accessing care in the public sector, with limited choice. Hence, the system design becomes crucial in the public sector medicines’ supply chain even more than in the private sector.

The list of essential medicines was reduced by retaining only common dosages required, removing combination drugs and non-generic medicines. The 287 medicines in the EDL were arrived upon by a committee of experts who were well versed with the disease burden of UP, comparison with the WHO essential medicines’ list and other states EDL and adapting it for local requirements, covering most of the patient requirements. Despite a significant reduction in the number of essential medicines, the procurement and consumption value have increased substantially.

Redesigning existing entities to adapt and adopt successfully was a complex effort. It required sustained support and leadership. GoUP’s commitment to having a robust public health supply chain played a crucial role. While TNMSC has been heavily relied on in the redesign of UPMSC, it is important to note the difference in scale and context between UP and Tamil Nadu. The population (240 million vs. 70 million), number of districts (75 vs. 38), state capacity, budgets, availability of technical resources, and varying health burden required UP-specific adaptations in the public health supply chain suited for its complexity. While TN started de-nova and could improve the supply chain model over a period, there was pressure on UP to show success early on. Hence, learning lessons from successful entities and adopting them for UP’s context with a staggered implementation plan was critical for success.

UPTSU learned and identified the seven essential pillars and their key processes for redesign and successful adaptation.

### 3.1 Seven essential pillars of the public health supply chain system

1. Essential Drug List (EDL) and Scientific Forecasting: In UP, the EDL was pruned down from ~1300 to ~300 items. Initial demand was forecasted based on end-user consumption data, disease patterns and demography of the state.
2. Warehouses: District Warehouses are critical cogs of the public health supply chain system. UPMSC controls these warehouses and is responsible for maintaining a 24/7 supply of all essential medicines at the warehouses. Only the medicines’ movement in and out of warehouses is captured in real time. The number of points to be monitored is manageable but critical. Only the medicines that are consumed (taken to facilities) are replenished in the warehouses, leading to a consumption-based ordering, which ensures that there is minimal wastage.
3. Centralized Procurement: Centralized procurement of medicines through rate contracting and issuance of centralized purchase orders. This helps in economies of scale and the maintenance of medicine availability across the state. Many vendors can also be pre-qualified based on inspections and past performance. UPMSC is also planning to monitor the performance of suppliers to build a database of credible suppliers to ease the transaction costs of suppliers in the future. The time period for the finalization of tenders can be reduced substantially.
4. The Passbook System: A passbook system has been established, using which each facility is responsible for picking up medicines as per their requirement within the budget allocated. The passbook system provides flexibility and visibility on medicines’ consumption patterns among health facilities, links high footfall facilities with medicines’ usage, forecasts medicines’ requirements, and prevents pilferage due to the double-entry system.
5. Quality Control: Each batch of medicines supplied at warehouses is tested by empaneled NABL-accredited laboratories to ensure the quality of the medicines. A “*Double Blind*” method is followed to ensure no single party knows details of both the information about the batch and the laboratory where the sample has been sent for testing. The blinding process involves removing the identifiers that can link the sample to the manufacturer or warehouses and adding a system-generated code. Samples from each of the warehouses where the medicines are supplied are sent to headquarters. The DVDMS performs two-step randomization, where it first randomly selects a sample that has been received and then randomly assigns an accredited quality control laboratory where the sample will be sent for testing. The medicines under testing are kept under the “quarantine” area and are released to public health facilities only after successful testing by the laboratories.
6. Centralized Payment: Centralized payments are being made to the vendors so that they are not required to follow up with the multiple district-level authorities for the release of their payment. However, there is further scope to reduce the delay in payments.
7. Horizontal cross-cutting-e-Tracking (DVDMS): DVDMS, an online logistics management information system for procurement and inventory management of medicines, serves as the IT backbone of UPMSC. It provides real-time data on stock inventory at the various warehouses and helps generate automatic placement of supply orders based on consumption of each EDL medicine from all the warehouses with a specific quantity per consignee (warehouse). This system can later be linked to the electronic health records systems of the hospitals to track end point consumption at the hospital level.

The legacy system of UP (CMSD) was not responsible for aspects of the supply chain such as indenting, supplier performance, quality assurance, and prompt payments. The legacy model had a fluid responsibility matrix. No one in the chain of command was responsible for vendor performance, which was critical for the availability of medicines. The quality control was also vendor-dependent, with no one responsible for ensuring that unsatisfactory medicines do not reach the facilities. The reasons for the non-availability of medicines at the facilities were tossed between CMSD, the vendor, the district officials, and the facility in charge, leading to a blame game. Even if a medicine is available, it is not necessarily required for patient care, and hence, facilities were often recipients of medicines in excess. Whereas, the ‘pull system’ model of UPMSC demarcates the responsibility matrix for medicines’ availability between the two major stakeholders—UPMSC, which is responsible for all essential medicines’ availability at the warehouses and facility in-charges, who are responsible for the availability of medicines required at the concerned facility.

The system design of UPMSC allows resources to be allocated based on real-time, ground-level needs instead of assumptions made at the top. This is done by two processes. The facilities have complete freedom to take medicines of their choice based on the needs of the patients they serve. This is reflected in the outgo from the warehouses based on real-time ground-level needs. The UPMSC only replenishes the outgo from the warehouses without any assumptions by placing monthly centralized indents to the suppliers based on actual consumption.

Though this paper documents the significant improvements in drug availability at the warehouses, purchase orders, and consumption values, and in the number of batches of medicines tested, it is difficult to establish causality between the reforms and process changes to the improvements mentioned above due to the absence of comparative data. However, there were no pandemic-driven reforms or external donor support in strengthening UPMSC, as the reforms happened prior to the pandemic and were government-driven. The state budget for medicines has increased from 74 million USD in 2017–2018 to 168 million USD in 2024–2025, indicating better capacity of UPMSC to procure and supply medicines due to the improved system design and process reforms. Even with clear data on the positive directions in the performance of UPMSC so far, sustained effort is required to reach the goal of ensuring 24×7 availability of medicines in 75 district warehouses, the transportation of medicines to the facilities, particularly to the 25,000 plus health and wellness centers. Quality assurance mechanisms need further strengthening to ensure all batches are tested in a timely manner and prompt system-based payments to vendors.

DVDMS flags expiry of medicines, batch recalls if any, and non-standard quality (NSQ) medicines. This ensures that only quality-assured medicines are released to facilities for consumption. The DVDMS system prevents pilferage by linking supplier dispatches with warehouses’ receipts (inbound) and linking warehouse medicines’ issuance with facility receipts (outbound). The real-time availability of medicines in warehouses is also displayed for public view in DVDMS. These measures improve transparency and accountability in the medicines’ supply chain. Efforts are also being undertaken to integrate the DVDMS system with e-hospital systems of the facilities to understand real-time availability of medicines at the last-mile and to capture medicines prescribed to the patients. At the community level, the adequate availability of medicines such as iron folic acid (IFA) for pregnant women and paracetamol syrup for children has also shown significant improvements. (Program data).

It is important to note that rental warehouses have their challenges in terms of infrastructure, such as cold chain, pest control, and fire safety requirements. Rental warehouses were always positioned as a transitionary provision until permanent warehouses under the control of UPMSC are built. Funds have been secured for all 75 districts, and construction is expected to be completed soon, overcoming this issue.

## 4 Limitations

This study has a few limitations. The study does not cover the larger ecosystem of the pharmaceutical industry’s capacity due to simultaneous orders from multiple states, patents, and its interactions with medicines’ access. EDL, in large part, are generic medicines and are not covered under the patent regime. The study does not cover cost comparison between the old and new systems in UP or in comparison with TNMSC. There is adequate literature on cost–benefit in procurement through the TNMSC model, and data from DVDMS suggest that the cost for most medicines is less in comparison with the CMSD model. The study does not provide data on the last-mile availability, as the DVDMS system captures only warehouse outgo. Field inspections reveal that medicines’ availability has improved at the end point, and the rollout of e-hospital systems will help capture this data. The change in prescription practices of doctors required to prescribe available EDL drugs is not part of this paper. The impact of OOP expenditure on the population level and the increase in the utilization of public health services due to UPMSC needs a separate study.

## 5 Conclusion

An explicit focus on medicines is necessary for health systems to achieve the goals of universal health coverage (21). While many states have visited TNMSC, few have been able to emulate the model successfully. We believe that the nuances of system design have not been fully understood, and much emphasis has been placed on contextual factors for success and failure. Many states have not followed the seven essential pillars and key processes. Redesign of the UPMSC supply chain model has led to significant improvements in the availability of essential drugs in 75 district warehouses of UP. The consistent availability of essential medicines in all the district warehouses and their ever-increasing consumption by the 30,000-plus public health facilities serve as a signal toward the availability of required medicines for free to the poor people who visit these facilities. Some of the states have added certain other functions, such as construction in the role of the procurement agency, reducing their razor-sharp focus on medicine availability. Some have found it difficult to ensure the prompt payment aspect and robust quality control mechanisms. UPMSC needs to strengthen commodities and equipment procurement, similar to that of the procurement of medicines. There is a need for GoUP to work on improving the prescription practices of doctors in the public health system to align with the essential drug list. Policy measures to reduce the influence of private pharmaceutical companies on the prescription practices of government doctors also need to be done. GoUP and UPTSU learned some of the key elements, system design, and processes based on the redesign experience of the public health supply chain system in UP that can help other states of India and other LMICs with a similar context.

## Data Availability

The original contributions presented in the study are included in the article, further inquiries can be directed to the corresponding author. Any unpublished documentation can be obtained from the corresponding author, upon reasonable request.

## Footnotes

^1^EY, Uttar Pradesh Health Systems Strengthening Project. Procurement and Supply Chain Gap Analysis Report, Report No: 2015- Delhi-0090, [unpublished report].

^2^Minutes of the meeting regarding ‘Strengthening of Public Health LMIS.

^3^Government Order. No 408 dated 13 March 2019 regarding mechanisms for establishment of warehouses, maintenance and distribution of drugs.
